# Dispersal strategies of phytophagous insects at a local scale: adaptive potential of aphids in an agricultural environment

**DOI:** 10.1186/1471-2148-6-75

**Published:** 2006-10-02

**Authors:** Eric Lombaert, Roger Boll, Laurent Lapchin

**Affiliations:** 1 Unité de Lutte Biologique, INRA, 06903 Sophia-Antipolis, France; 2 UMR ROSE, INRA-UNSA, 06903 Sophia-Antipolis, France

## Abstract

**Background:**

The spread of agriculture greatly modified the selective pressures exerted by plants on phytophagous insects, by providing these insects with a high-level resource, structured in time and space. The life history, behavioural and physiological traits of some insect species may have evolved in response to these changes, allowing them to crowd on crops and to become agricultural pests. Dispersal, which is one of these traits, is a key concept in evolutionary biology but has been over-simplified in most theoretical studies. We evaluated the impact of the local-scale dispersal strategy of phytophagous insects on their fitness, using an individual-based model to simulate population dynamics and dispersal between leaves and plants, by walking and flying, of the aphid *Aphis gossypii*, a major agricultural pest, in a melon field. We compared the optimal values for dispersal parameters in the model with the corresponding observed values in experimental trials.

**Results:**

We show that the rates of walking and flying disperser production on leaves were the most important traits determining the fitness criteria, whereas dispersal distance and the clustering of flying dispersers on the target plant had no effect. We further show that the effect of dispersal parameters on aphid fitness depended strongly on plant characteristics.

**Conclusion:**

Parameters defining the dispersal strategies of aphids at a local scale are key components of the fitness of these insects and may thus be essential in the adaptation to agricultural environments that are structured in space and time. Moreover, the fact that the effect of dispersal parameters on aphid fitness depends strongly on plant characteristics suggests that traits defining aphid dispersal strategies may be a cornerstone of host-plant specialization.

## Background

Dispersal is a key concept in evolutionary biology. Surprisingly, traits describing dispersal strategies are over-simplified in most theoretical studies [1], with the exception of behavioral traits [2]. Optimal foraging theory [3] takes into account the movements of animals searching for resources, but even in this case, models of the evolution of behavioral traits are rarely associated with complex ecological situations [but see [4,5]]. However, the ability to disperse at different spatial scales probably makes a major contribution to the fitness of individuals in a given resource structure [6] and this relationship presumably depends on the trade-off between costly, risky dispersal and local competition [7]. Phytophagous insects are subject to many selective pressures, including the abundance and diversity of plants and their spatial and temporal fluctuations. The spread of agriculture greatly modified the pattern of resources of phytophagous insects [see for example [8]]. At a local scale, crop plants are usually highly homogeneous in terms of spatial distribution and quality, and provide a high-level resource for consumers. At a larger scale, cultivated plant species are distributed in patches of variable size. Moreover, for most crops, these resources are available for only a limited period of the year. Agricultural development is therefore thought to favor phytophagous insects with a high rate of increase on a plant to which they are specialized and with dispersal strategies adapted to both the optimal exploitation of locally abundant resources and the colonization of fluctuating large-scale agricultural metapopulation landscapes.

Most aphid species combine a high rate of increase and efficient dispersal. The combination of a short development time and clonal reproduction leads to local population explosions and strong kin competition [9,10]. The medium- and large-scale dispersal of aphids is favored by polyphenism: apterous and winged aphids are produced simultaneously by a given clonal population [11,12]. Aphids were thus good candidates to become major pests when agricultural systems were first developed, in terms of both local dynamics and large-scale dispersal. They have indeed become worldwide major pests of many commercial crops [13]. Most studies on aphid dispersal have focused on large-scale movements [14] and little is known about whether local dispersal strategies are well adapted to agriculture expansion. The exponential growth of the population observed in environmental conditions is fuelled largely by an increase in the number of apterous individuals, which were long considered sedentary. However, apterous individuals are also involved in small-scale dispersal, walking from leaf to leaf or from one plant to a neighboring plant [15,16]. Aphid dispersal thus combines nested strategies, each of which is associated with various possible costs: the first strategy level is to invest or not into dispersal with the respective risks of failing to find a new profitable environment or being limited by local resource depletion. The next level in the dispersal strategy concerns whether or not to produce winged offspring. Winged individuals increase dispersal capacity in terms of the distance that can be covered, but there is a trade-off between wing muscle production and reproductive investment [17-19]. The third level of the dispersal strategy concerns the kind of flight to be adopted by a winged aphid: a costly migratory flight or a trivial flight which is less risky and less efficient at reducing intraspecific competition [20,21].

We analyzed the dispersal strategies of the melon aphid *Aphis gossypii *on cultivated melon plants (*Cucumis melo*) at the leaf and field scales. *Aphis gossypii *displays strictly clonal reproduction in Europe and is one of the main arthropod pests and virus vectors of diverse, economically important crops such as cotton, curcurbits and citrus plants. *A. gossypii *multiplies rapidly in favorable conditions, providing logistic population growth [9] with strongly density-dependent dispersal polyphenism [22-24]. *A. gossypii *thus displays most of the phenotypic traits favoring fitness in agricultural environments. We explored the adaptive goodness of fit of these local-scale dispersal strategies in an agricultural structure, by means of an individual-based simulation model. Parameters were estimated using two greenhouse experiments. The results obtained confirm that the parameters defining the dispersal strategies of aphids at a local scale are well adapted to agricultural environments. They demonstrate that the rates of walking and flying disperser production at the leaf scale are key components of aphid fitness and that optimal fitness depends strongly on resource structure. This suggests that traits defining aphid dispersal strategies may be a cornerstone of host-plant specialization.

## Results

### Observed dispersal parameters

**Figure 1:**
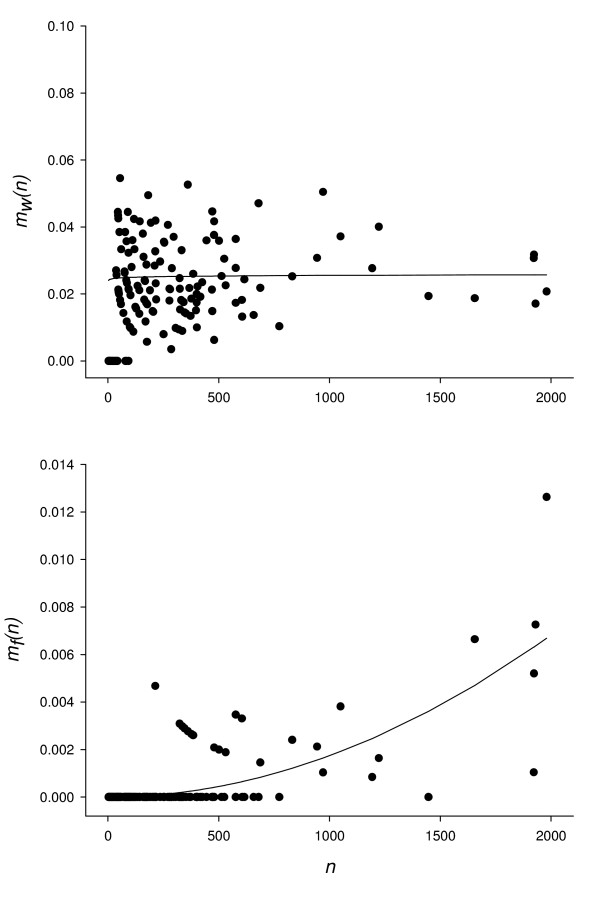
**Observed leaf-scale dispersal rates**. Observed leaf-scale dispersal rates (points) of walking dispersers *m*_*w*_*(n) *and flying dispersers *m*_*f*_*(n) *during experiment 1, expressed in terms of the total number of aphids on a given leaf *n*. The line shows the values predicted by nonlinear regression analysis (see methods).

From Experiment 1 (see methods), estimated values of dispersal rate parameters were *A*_*w *_= 0.0238 and *B*_*w *_= 0.01 for walking dispersers (*F*_*2, 165 *_= 597.66; *P *< 10^-4^), and *A*_*f *_= 2.16E^-9 ^and *B*_*f *_= 1.97 for flying dispersers (*F*_*2, 165 *_= 387.71; *P *< 10^-4^) (Fig. 1). The exponent parameter *B*_*w *_was not significantly different from 0 (95% CI: [-0.07; 0.09]). The rate *m*_*w*_*(n) *(see methods, eq. 4) can thus be considered to be independent of aphid density. In contrast, the rate *m*_*f*_*(n) *(see methods, eq. 5) increased strongly with the number of aphids on the source plant. We compared the distribution of dispersing apterous aphids on the target leaves with a uniform distribution in which half the aphids settled on each of the two leaves, by carrying out a Chi-squared test for every day and plant with more than 20 apterous dispersers. Eleven times in twelve, the numbers of aphids on the upper and lower target leaves differed significantly, with a 5% type 1 error threshold. Moreover, there were always more aphids moving towards the third leaf than towards the first leaf (means of 82 % vs. 18 %; sign test: *P *< 0.0005). Thus, the dispersal of walking aphids was preferentially towards the younger of the two target leaves.

**Figure 2:**
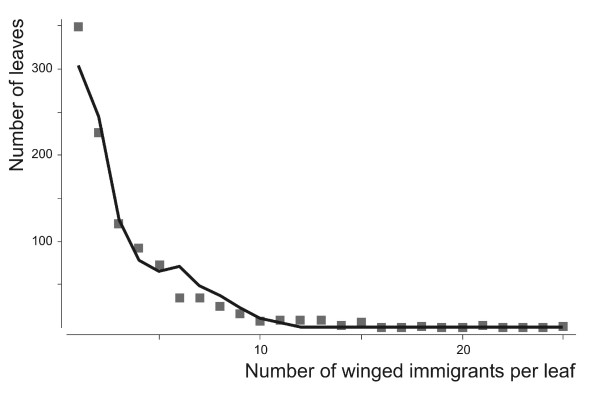
**Frequency distribution of winged aphids on leaves of target plants**. Observed (points) and simulated (line) frequency distribution of winged aphids landing on the leaves of neighboring plants, with the aggregation parameter *a *and the frequency of attractive leaves *f*_*at *_set to 6 and 0.35, respectively.

From experiment 2 (see methods), the estimated value of *r*_0 _was 0.37 day^-1 ^(*F*_2, 17 _= 3005.68; *P *< 10^-4^). We studied the spatial distribution of winged aphids landing on the neighboring plants by using Chi-squared tests to compare the number of winged aphids collected daily from each leaf of the target plants with a random distribution, predicted from a Poisson distribution. No aphid was found on the target plants before day 18, when the total number of aphids on the source plant was about 6800. From day 18 onwards, the daily flow of aphids to the target plants increased exponentially. Most of the aphids collected from the target plants were winged, but very rare apterous individuals were also involved in colonization. The hypothesis of a random distribution was rejected for all days analyzed (*P *< 10^-3 ^for each day tested, i.e. the five last days of the experiment). The variance was always higher than the mean, suggesting that the individuals tended to clump within their environment. We investigated the aggregation pattern of the winged aphids landing on the leaves, by comparing the experimental data with the results of simulations using sets of values of *f*_*at *_from 0 to 1 and of *a *from 1 to 100. For each combination of these two parameters, we calculated the maximum difference between the cumulative frequencies of the observed and simulated distributions. The lowest cumulative frequency difference was obtained for *f*_*at *_= 0.35 and *a = 6 *(Fig. 2).

### Simulation results

**Figure 3:**
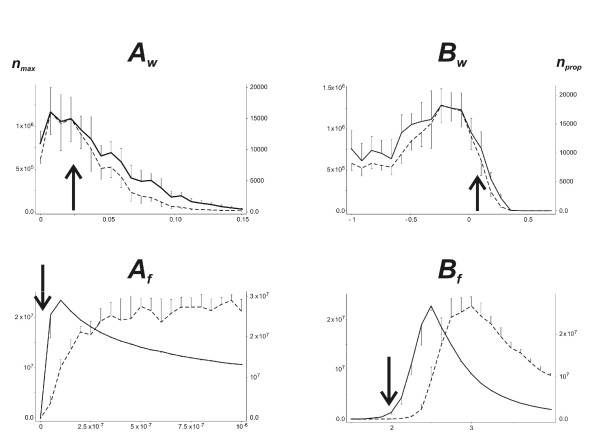
**Effect of dispersal parameters on fitness criteria**. Effect of varying walking (*A*_*w*_, *B*_*w*_) and flying (*A*_*f*_, *B*_*f*_) disperser aphid production parameters on the maximum number of aphids observed in the greenhouse (*n*_*max*_, solid lines) and the production of propagules (*n*_*prop*_, doted lines). Arrows indicate the experimentally observed values of the parameters. Half error bars represent the standard deviation obtained from the 10 simulations of each point.

Fig. 3 shows the values of the two fitness criteria, *n*_*max *_and *n*_*prop *_(see methods), when each of the four dispersal parameters were varied individually, the other three parameters in each case being set to the values estimated from Experiment 1. Sets of 10 simulations were run for each of 21 values from 0 to 0.15 for *A*_*w*_, from -1 to 0.7 for *B*_*w*_, from 0 to 10^-6 ^for *A*_*f *_and from 1.5 to 4 for *B*_*f*_. Both optimality criteria were maximal for a range of values of *A*_*w *_between 0.005 and 0.03 when the observed value was 0.0238. For *B*_*w*_, both criteria were maximal for a range of values between -0.5 and 0.1 when the observed value was 0.01. *n*_*max *_increased sharply then gradually decreased for values of *A*_*f *_above 5E^-8^, and *n*_*prop *_increased continuously with *A*_*f *_when the observed value was 2.16E^-9^. A maximum of *n*_*max *_was observed for *B*_*f *_= 2.4 and a maximum of *n*_*prop *_was obtained with *B*_*f *_= 3 when the observed value was 1.97. The observed values for the dispersal parameters of walking individuals were thus compatible with the optimal fitness calculated with the simulation model. In contrast, the observed dispersal parameters of flying individuals gave calculated fitness parameters far lower than the theoretical maximum.

**Figure 4:**
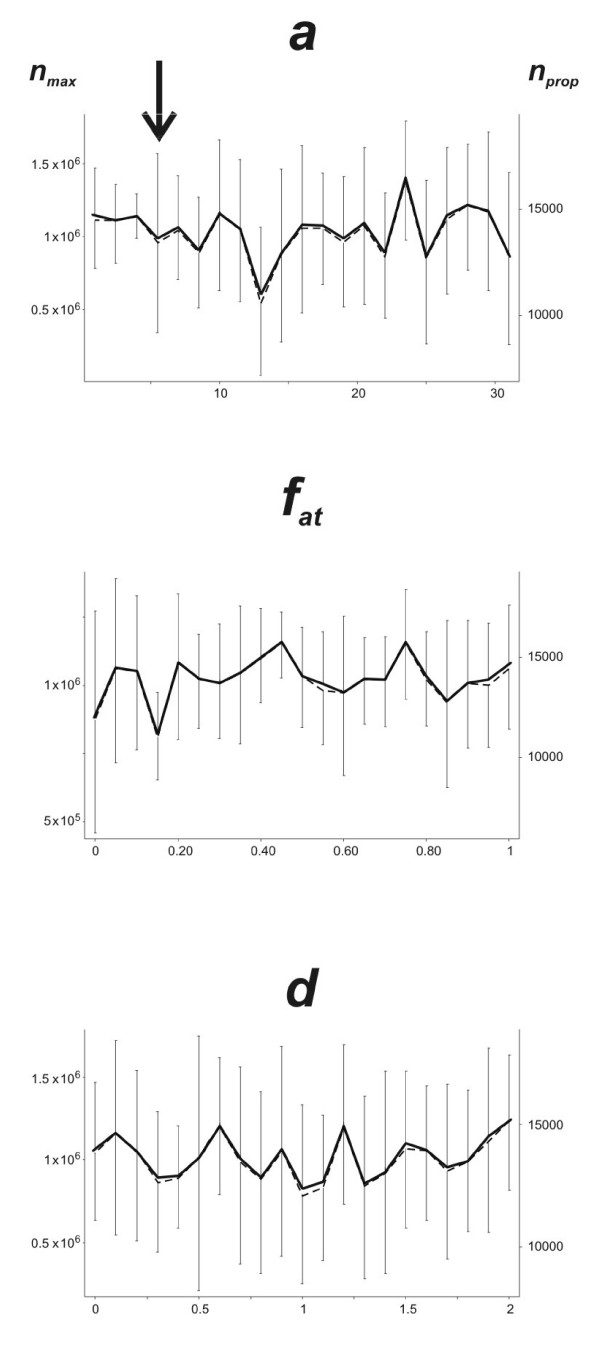
**Effect of aggregation parameters on fitness criteria**. Effect of varying the aggregation parameters of winged aphids on their target leaves (*a*, *f*_*at*_) and the parameter limiting the flying distance (*d*) on the maximum number of aphids observed in the greenhouse (*n*_*max*_, solid lines) and the production of propagules (*n*_*prop*_, doted lines). Arrows indicate the experimentally observed values of the aggregation parameters. Half error bars represent the standard deviation obtained from the 10 simulations of each point.

The effects of differential leaf attractivity and dispersal distance are shown in Fig. 4. They clearly have no effect on fitness criteria *n*_*max *_and *n*_*prop*_.

**Figure 5:**
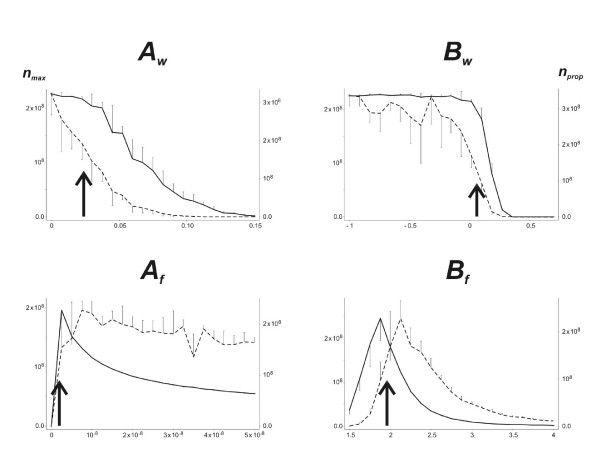
**Effect of dispersal parameters on fitness criteria in the case of a cucumber crop**. Changing the plant parameters: effect of varying walking (*A*_*w*_, *B*_*w*_) and flying (*A*_*f*_, *B*_*f*_) disperser aphid production parameters on the maximum number of aphids observed in the greenhouse (*n*_*max*_, solid lines) and the production of propagules (*n*_*prop*_, doted lines) in the case of a cucumber crop. Arrows indicate the experimentally observed values of the parameters in a melon crop. Half error bars represent the standard deviation obtained from the 10 simulations of each point.

## Discussion

Experiments and simulation models confirmed that the behavioral and polyphenism traits defining the dispersal strategies of a given aphid clone at a local scale strongly influence the fitness of that clone in agricultural conditions. These relationships between aphid dispersal strategies and fitness raise two types of question. The first concerns the optimal dispersal rules minimizing kin competition in a given environment. The second concerns the goodness of fit between aphid dispersal rules and resource characteristics, which is likely to affect the host plant specialization of aphids.

Our simulations demonstrated that the parameters determining the rates of production of walking and flying dispersers and their density dependence have a crucial impact on aphid fitness, expressed in terms of insect density in the field and in the production of potential propagules able to colonize other fields. Conversely, the dispersal distance of flying aphids at the field scale and their aggregation on target leaves have no effect on aphid fitness. The observed parameters for walking disperser production on melon plants were close to the optimal values determined by modeling, even though observed values were roughly estimated from field experiment. In contrast, the parameters for flying disperser production gave fitness estimates far lower than the predicted maximum.

Two important parameters were fixed in the simulation models. The "sedentarization rate" was arbitrary set to 0.35. Complementary sets of simulations were run with *s*_*r *_= 0.15, 0.25, 045 or 055. The curves obtained with these values (data not shown) were roughly homothetic from the curves of Fig. 3, indicating that the qualitative results are not modified by the sedentarization rate. The sensitivity of the model results to the value of the maximum potential rate of increase *r*_0 _= 0.37 calculated from field data was also evaluated, using *r*_0 _= 0.27, 0.32, 0.42 or 0.47. Again, the resulting curves were homothetic. The optimality of the dispersal parameters was not influenced by *s*_*r *_or *r*_0_.

### When should aphids leave the colony?

The observed and predicted rates of walking disperser production were density-independent; the constant rate of walking disperser production was low (about 2%), but played an important role in local population dynamics. However, the abundance of walking individuals may increase strongly in cases of sudden stress affecting the source plant [25]. The between-leaf dispersal of walking aphids began very early and mostly involved adults [as seen by [26]]. In Experiment 1, walking dispersers showed a strong preference for colonization of the leaf above the original leaf rather than the leaf below. As melon plants have a sprawling growth habit, this choice is probably related to sap testing rather than to negative geotropism. This preference also increased the probability of colonizing newly produced leaves, thereby avoiding kin competition. Local dispersal therefore made it possible to exploit fully the new leaf area exposed as a result of intensive production.

In contrast, the production of flying individuals to undertake more risky aerial dispersal was strongly density-dependent and involved a large proportion of the population only when local crowding increased dramatically, as previously reported for other aphid species [11,16,20,27,28].

Thus, the morphological and behavioral mechanisms underlying the departure rules of *A. gossypii *make it possible for the aphids to take advantage of high-level resources in plant crops: fast-growing colonies first optimize leaf area occupancy, with a constant proportion of walking dispersers able to cover short distances. Winged aphids are then produced at a higher rate, dependent on density at the leaf scale. The leaf resource is thus maximally exploited and the combination of population dynamics on leaves and of progressive leaf contamination leads to a gradual increase in the flux of winged aphids at the plant scale, resulting in colonization over greater distances.

### Where should the aphids go?

The parameters determining the timing of departure from the source plant are of prime importance in aphid fitness in agricultural conditions. Surprisingly, we found that the parameters determining the site to which aphids moved in the field had no impact, as demonstrated by modeling, although we did not experimentally evaluate the distance covered by winged dispersers within the field. However, the model took into account the indirect costs of dispersal due to kin competition, but not the direct costs of moving (i.e. energy and time consumption), which generally increase with distance [29-33]. However, these costs are probably negligible at the field scale.

In other respects, it could be predicted that optimal dispersal strategy at a medium-scale should tend towards a uniform distribution of winged colonizers: *Aphis gossypii *reproduces by thelytokous parthenogenesis and individuals of this species therefore do not need to find mates for sexual reproduction [34]. Moreover, the logistic shape of population growth curve suggests a high level of competition between disperser offspring. In our experiments, winged individuals clearly tended to clump together on the various leaves of the target plants (Experiment 2 – Fig. 2). This trend has been reported in several aphid species [24,35-37]. It may be adaptive in wild environments, where resource plants are rare and sparse. In such cases, as dispersal by flying is risky [38,39], the chances of success may be higher if winged aphids are attracted by the presence of congeners or by their effect on the host plant. Moreover, for some animal species, such as some birds, fish and herbivorous mammals, clumping provides protection against predation [40]. Such benefits of aggregation have also been observed in insect species, including aphids [41-44].

Therefore, the behavioral rules governing landing by winged *A. gossypii *seem to be poorly adapted to spatially uniform and high-level resources, if other potential selective pressures, such as natural enemies, are not considered. However, simulations demonstrated that the aggregation of winged dispersers had no effect on fitness criteria, even when aggregation parameters were set to values resulting in stronger aggregation than observed in our experiments. Apparently poorly adaptive landing traits may thus be of minor importance for aphid fitness in the agricultural environment.

### Local dispersal traits and aphid specialization on host plant species

When considering the discrepancy between observed and optimal values of the parameters of flying disperser production, it should be borne in mind that the NM1 clone of *A. gossypii *is specialized for host plants of the cucurbit family [45,46]. Cultivated species of this family, such as zucchini, cucumber and melon plants, grow rapidly and have a large carrying capacity for *A. gossypii*, which is the only aphid pest of these crops. However, this carrying capacity differs considerably between the plant species of this family, with large cucumber leaves able to support more than 10,000 aphids each [47]. We roughly evaluated the optimality of disperser production parameters for cucumber plants by running sets of simulations similar to those for melon plants, but setting the leaf carrying capacity parameter *K *to 70,000 and the resource depletion parameter *KK *to 5 (see methods, eq. 3). These values produced a population dynamics curve at the leaf scale similar to the previous curve, but with a maximum number of aphids close to 10,000 (data not shown). As shown in Fig. 3 for simulated melon crops, Fig. 5 shows the values of both fitness criteria when each of the four dispersal parameters were varied individually, the other three parameters being set to the values estimated from Experiment 1. The optimal values of the parameters for walking disperser production in melon crops were also almost optimal for cucumber crops, except that walking dispersal conferred neither an advantage nor a disadvantage for clonal fitness: neither of these fitness criteria was lower for *A*_*w *_= 0 than for *A*_*w *_> 0. However, the observed values of *A*_*f *_and *B*_*f*_, which were far from optimal for flying disperser production in melon crops, were almost optimal for cucumber. Moreover, whatever the parameter values used, the "cucumber" simulations gave much larger numbers of aphids and propagules than the "melon" simulations. This suggests that: (1) the disperser production traits of genotypes of *A. gossypii *specialized on cucurbit plants are adapted to the host plant, increasing aphid fitness, as on cucumber plants, in terms of local density or large-scale dispersal; and (2) the parameters with the greatest power for selection are those determining the density-dependent production of flying dispersers. Moderate, density-independent walking dispersal may confer a selective advantage when the resource is not optimal.

## Conclusion

Our results show that local dispersal strategies have a strong impact on fitness and are thus very important from an evolutionary point of view. The recent adaptation of phytophagous insect to agriculture cannot be explained solely by adaptive responses to pest control tools such as biological control, pesticides or resistant cultivars. The potential to make use of a temporary, high-level resource, homogeneous on a medium spatial scale and fragmented on a large scale, must also be taken into account [48]. Our work also highlights the importance of taking into account local dispersal strategies in studies of population dynamics on larger scales [49-52]. It suggests that some arthropod pests, such as aphids, displaying very high rates of population increase and combining different dispersal strategies at different scales, may adapt more easily to this new type of environment than other phytophagous organisms. The fitness of such populations seems to be highly sensitive to dispersal parameter values. This also suggests that different dispersal strategies may have been selected in the different biotypes *of Aphis gossypii*, which are specialized on different host plants, with various types of spatial structure and carrying capacity. As aphid specialization seems to occur at the host plant family or tribe level [8,45,46], the precise tuning of population dynamics parameters on a preferred and abundant resource species may lead to a phytophagous insect species becoming a major pest on this species but less detrimental on other crops of the same family. Exploration of the genetic variability associated with dispersal strategies [23,53] is required to confirm that these traits are subject to natural selection and to determine whether dispersal strategies could be affected by the costs of selective responses to crop protection methods, such as pesticide resistance or overcoming the resistance of plant cultivars.

## Methods

### The model

We constructed an individual-based model to simulate aphid population dynamics on melon plants and to explore optimal dispersal patterns at the field scale. Each crop consisted of a 10 × 10 grid of regularly spaced plants, studied for 70 days (approximate length of the melon crop cycle in fields in Southern France). We assumed that leaves were present on only one stem per plant. The initial plant stage was set at five leaves and plant growth was simulated using Verhulst's logistic expression of the number *l*_*j *_of leaves on day *j *per plant:

lj=lmax⁡⋅l0erl⋅jlmax⁡+l0⋅(en⋅j−1)     (1)
 MathType@MTEF@5@5@+=feaafiart1ev1aaatCvAUfKttLearuWrP9MDH5MBPbIqV92AaeXatLxBI9gBaebbnrfifHhDYfgasaacH8akY=wiFfYdH8Gipec8Eeeu0xXdbba9frFj0=OqFfea0dXdd9vqai=hGuQ8kuc9pgc9s8qqaq=dirpe0xb9q8qiLsFr0=vr0=vr0dc8meaabaqaciaacaGaaeqabaqabeGadaaakeaacqWGSbaBdaWgaaWcbaGaemOAaOgabeaakiabg2da9iabdYgaSnaaBaaaleaacyGGTbqBcqGGHbqycqGG4baEaeqaaOGaeyyXICTaemiBaW2aaSbaaSqaaiabicdaWaqabaGcdaWcaaqaaiabdwgaLnaaCaaaleqabaGaemOCai3aaSbaaWqaaiabdYgaSbqabaWccqGHflY1cqWGQbGAaaaakeaacqWGSbaBdaWgaaWcbaGagiyBa0MaeiyyaeMaeiiEaGhabeaakiabgUcaRiabdYgaSnaaBaaaleaacqaIWaamaeqaaOGaeyyXIC9aaeWaceaacqWGLbqzdaahaaWcbeqaaiabd6gaUjabgwSixlabdQgaQbaakiabgkHiTiabigdaXaGaayjkaiaawMcaaaaacaWLjaGaaCzcamaabmGabaGaeGymaedacaGLOaGaayzkaaaaaa@5CA4@

where *r*_*l *_is the daily rate of increase in the number of leaves and *l*_*max *_the maximum number of leaves per plant.

At the beginning of the simulation, one aphid was randomly installed on one leaf of the crop. Aphid dynamics on a single leaf took into account the maximum potential rate of increase in aphid numbers, a theoretical carrying capacity of the leaf, variation of this carrying capacity according to leaf resource depletion, and dispersal by apterous or winged individuals. As the number of aphids on each leaf may also be modified by daily colonization, a continuous equation was not appropriate and a recurrence formula derived from the Ricker's logistic model was used:

*n*_*j*+1 _= *n*_*j*_·*e*^*r *^      (2)

with:

r=r0⋅(1−njK−cnjK⋅KK)−mw(nj)−mf(nj)     (3)
 MathType@MTEF@5@5@+=feaafiart1ev1aaatCvAUfKttLearuWrP9MDH5MBPbIqV92AaeXatLxBI9gBaebbnrfifHhDYfgasaacH8akY=wiFfYdH8Gipec8Eeeu0xXdbba9frFj0=OqFfea0dXdd9vqai=hGuQ8kuc9pgc9s8qqaq=dirpe0xb9q8qiLsFr0=vr0=vr0dc8meaabaqaciaacaGaaeqabaqabeGadaaakeaacqWGYbGCcqGH9aqpcqWGYbGCdaWgaaWcbaGaeGimaadabeaakiabgwSixpaabmGabaGaeGymaeJaeyOeI0YaaSaaaeaacqWGUbGBdaWgaaWcbaGaemOAaOgabeaaaOqaaiabdUealbaacqGHsisldaWcaaqaaiabdogaJjabd6gaUnaaBaaaleaacqWGQbGAaeqaaaGcbaGaem4saSKaeyyXICTaem4saSKaem4saSeaaaGaayjkaiaawMcaaiabgkHiTiabd2gaTnaaBaaaleaacqWG3bWDaeqaaOWaaeWaceaacqWGUbGBdaWgaaWcbaGaemOAaOgabeaaaOGaayjkaiaawMcaaiabgkHiTiabd2gaTnaaBaaaleaacqWGMbGzaeqaaOWaaeWaceaacqWGUbGBdaWgaaWcbaGaemOAaOgabeaaaOGaayjkaiaawMcaaiaaxMaacaWLjaWaaeWaceaacqaIZaWmaiaawIcacaGLPaaaaaa@5B15@

where *r *is the real rate of increase in aphid numbers, *r*_0 _the maximum potential rate of increase, *n*_*j *_the number of aphids on the leaf on day *j*, *cn*_*j *_the cumulative number of aphids on the leaf on day *j, K *the potential carrying capacity of one leaf, which together with *KK*, the ressource depletion parameter, defines the maximum cumulative number of aphids per leaf. We simulated integer numbers of aphids, by randomly assigning *n*_*j*_, after evaluation, to the closest integer value below or above the absolute number, with probabilities proportional to the fractional part of *n*_*j *_and to the complement to 1 of the fractional part of *n*_*j*_, respectively.

Dispersal rates were described by the following power functions:

mw(n)=Aw⋅nBw     (4)
 MathType@MTEF@5@5@+=feaafiart1ev1aaatCvAUfKttLearuWrP9MDH5MBPbIqV92AaeXatLxBI9gBaebbnrfifHhDYfgasaacH8akY=wiFfYdH8Gipec8Eeeu0xXdbba9frFj0=OqFfea0dXdd9vqai=hGuQ8kuc9pgc9s8qqaq=dirpe0xb9q8qiLsFr0=vr0=vr0dc8meaabaqaciaacaGaaeqabaqabeGadaaakeaacqWGTbqBdaWgaaWcbaGaem4DaChabeaakmaabmGabaGaemOBa4gacaGLOaGaayzkaaGaeyypa0Jaemyqae0aaSbaaSqaaiabdEha3bqabaGccqGHflY1cqWGUbGBdaahaaWcbeqaaiabdkeacnaaBaaameaacqWG3bWDaeqaaaaakiaaxMaacaWLjaWaaeWaceaacqaI0aanaiaawIcacaGLPaaaaaa@40C6@

for walking dispersers and

mf(n)=Af⋅nBf     (5)
 MathType@MTEF@5@5@+=feaafiart1ev1aaatCvAUfKttLearuWrP9MDH5MBPbIqV92AaeXatLxBI9gBaebbnrfifHhDYfgasaacH8akY=wiFfYdH8Gipec8Eeeu0xXdbba9frFj0=OqFfea0dXdd9vqai=hGuQ8kuc9pgc9s8qqaq=dirpe0xb9q8qiLsFr0=vr0=vr0dc8meaabaqaciaacaGaaeqabaqabeGadaaakeaacqWGTbqBdaWgaaWcbaGaemOzaygabeaakmaabmGabaGaemOBa4gacaGLOaGaayzkaaGaeyypa0Jaemyqae0aaSbaaSqaaiabdAgaMbqabaGccqGHflY1cqWGUbGBdaahaaWcbeqaaiabdkeacnaaBaaameaacqWGMbGzaeqaaaaakiaaxMaacaWLjaWaaeWaceaacqaI1aqnaiaawIcacaGLPaaaaaa@4062@

for flying dispersers.

In Experiment 1 (see below), plants were cut above the 3^rd ^leaf. We were thus unable to detail the aphid dispersal at the within plant scale. However, we used the results of this experiment to roughly approximate walking dispersal parameter values. Thus, we assumed that walking dispersers settled on the closest leaf below the source leaf, on the closest leaf above the source one and on the next leaf up with respective probabilities of 0.1, 0.8 and 0.1. If the source leaf was the lowest leaf of the plant, we assumed that walking dispersers settled on the two next leaves above the source with respective probabilities of 0.8 and 0.2. If the source leaf was the penultimate leaf, we assumed that walking dispersers settled on the leaf below with a probability of 0.2 and on the leaf above (the final leaf) with a probability of 0.8. Finally, if the source leaf was the top leaf of the plant, we assumed that walking dispersers could settle only on the leaf immediately below.

A "sedentarization rate" *s*_*r *_was applied to flying dispersers produced on the source leaf, to distinguish between winged aphids that successfully landed on a plant in the same field and aphids that died or dispersed out of the field. Winged aphids landing in the field were randomly distributed on the leaves of the plants. However, a non random landing pattern was also tested, to evaluate the possible effects of two factors: (1) landing being dependent on the distance between the source and target plants and (2) landing being aggregative, with some leaves more attractive than others.

The probability of landing on a plant located at a distance *D *from the source plant was proportionnal to:

*e*^-*d.D *^      (6)

where *D *is expressed in plant row or column number within the field. Dispersal distance decreases with increasing *d*, and most winged aphids land no further than the neighboring plants when *d *= 2. Conversely, winged dispersers are randomly distributed in the field when *d *= 0. As distance affects only the probability of a winged aphid landing on a given plant in the field, there is no border effect.

The "attractivity" effect was investigated by randomly defining a proportion *f*_*at *_of the leaves opened each day as "attractive". A coefficient *a *was assigned to the attractive leaves and the probabilities of landing on attractive and non-attractive leaves were proportionnal to *a *and 1, respectively.

### Evaluation of parameters

We set *r*_*l *_and *l*_*max *_to 0.1 and 200, respectively, to give a plant growth curve similar to that observed in the field (R. Boll, unpublished data). *K *was set to 3000 and *KK *to 17 to generate a population dynamics curve for a given leaf similar to that observed in the field (R. Boll, unpublished data), with a maximum number of aphids per leaf lower than 3000 and a gradual decrease in the number of aphids after this maximum had been reached. It was impossible to estimate the sedentarization rate *s*_*r *_from field experiments, because such estimates would have required replicates of precise counts of aphids present on each leaf of a source plant and counts of winged individuals landing on target plants and on the source plant. No quantitative data on winged aphid sedentarization rates are available from previous studies [but see [54]]. A set of simulations, using different values of *s*_*r*_, was first run and a value of *s*_*r *_= 0.35, was finally used for subsequent simulations.

Values of *r*_0_, *A*_*w*_, *B*_*w*_, *A*_*f*_, *B*_*f*_, *f*_*at *_and *a *for melon crops were estimated from two field experiments carried out in greenhouse conditions, for the description of strategies of aphid dispersal between leaves and between plants The aphid clone used in all experiments was NM1, which has been reared on melon plants in the laboratory since 1988. Before the experiments, NM1 was maintained in controlled conditions (20°C; L:D 16:8). The plant used (for insect rearing and for the experiments) was *Cucumis melo *cv. "Vedrantais". Plants were reared in a different insect-proof greenhouse from that used for the experiments. The mean temperature was about 30°C (ranging from 18°C to 45°C) for the first experiment, and about 25°C (ranging from 15°C to 40°C) for the second experiment. All statistical analyses were performed with SAS software [55].

#### Experiment 1: Between-leaf dispersal of Aphis gossypii

The aims of this experiment were: (1) to determine the dispersal rates of walking and flying aphids at the leaf scale and (2) to assess the local direction of walking aphids during dispersal. Ten melon seedlings were planted individually in 3-liter plant pots. The foliar structure of the plants was simplified: the stem was cut above the third leaf and all axillaries were systematically severed every day. When plants reached the three-leaf stage, the second leaf of each plant was infested by placing a single adult female on its abaxial surface. The second leaf was thus the source leaf and the first (lower; target leaf 1) and third (upper; target leaf 3) leaves were the target leaves. Each plant was placed in a Plexiglas cage. Every day, the total number of aphids on the source leaf was counted. All the winged and apterous adults and larvae located elsewhere were counted and removed and their positions were recorded: target leaf one, target leaf three, stem or off the plant. The total number of aphids *n *on the source leaf was determined each day. The rate of dispersal of walking individuals *m*_*w*_*(n)*, was calculated as *x*_*w*_*/n*, where, on a given day and in a given cage, *x*_*w *_is the number of walking dispersers found on the target leaves, and n is the total number of aphids on the source leaf. For flying dispersers, *m*_*f*_*(n) *was calculated as the ratio *x*_*f*_*/n*, where *x*_*f *_is the total number of winged dispersers found away from the source leaf. Winged individuals found on the target leaves were considered to be flying dispersers. The dispersal parameters of eq. 4 and 5 were calculated by nonlinear regression, using the NLIN procedure (Gauss-Newton method). As the first adults appeared on the fifth day and as less than 1% of walking dispersers were larvae, we excluded the first four days from analyses. The experiment was stopped 26 days after infestation.

#### Experiment 2: Between-plant dispersal of Aphis gossypii

The aim of this second experiment was (1) to estimate the maximum potential rate of increase of an aphid colony and (2) to study the spatial distribution of winged aphids landing on the neighboring plants. One aphid-infested melon plant (the source plant) was placed in the center of a 2 m × 2 m square containing eight aphid-free plants (target plants) of the same age. As the plants grew, main stems were rolled up so as to avoid contact between plants. Each plant was at the five-leaf stage at the beginning of the experiment. The second leaf of the source plant was infested with a small population of aphids. This population consisted of one adult female, three fourth instar larvae, three third instar larvae, four second instar larvae and four first instar larvae, to prevent demographic jolt and to facilitate estimation of the maximum potential rate of increase. The number of aphids on each leaf of the source plant was determined daily, either exhaustively, or with the help of visual abundance classes, which make it possible to obtain a rapid, reliable estimate of the real number of aphids on the plant [56]. The NLIN procedure was used to estimate the maximum potential rate of increase *r*_0 _(see eq. 3) on the source plant, according to a logistic growth model based on Verhulst's equation:

Nj=KpN0er0jKp+N0(er0j−1)     (7)
 MathType@MTEF@5@5@+=feaafiart1ev1aaatCvAUfKttLearuWrP9MDH5MBPbIqV92AaeXatLxBI9gBaebbnrfifHhDYfgasaacH8akY=wiFfYdH8Gipec8Eeeu0xXdbba9frFj0=OqFfea0dXdd9vqai=hGuQ8kuc9pgc9s8qqaq=dirpe0xb9q8qiLsFr0=vr0=vr0dc8meaabaqaciaacaGaaeqabaqabeGadaaakeaacqWGobGtdaWgaaWcbaGaemOAaOgabeaakiabg2da9maalaaabaGaem4saS0aaSbaaSqaaiabdchaWbqabaGccqWGobGtdaWgaaWcbaGaeGimaadabeaakiabdwgaLnaaCaaaleqabaGaemOCai3aaSbaaWqaaiabicdaWaqabaWccqWGQbGAaaaakeaacqWGlbWsdaWgaaWcbaGaemiCaahabeaakiabgUcaRiabd6eaonaaBaaaleaacqaIWaamaeqaaOWaaeWaceaacqWGLbqzdaahaaWcbeqaaiabdkhaYnaaBaaameaacqaIWaamaeqaaSGaemOAaOgaaOGaeyOeI0IaeGymaedacaGLOaGaayzkaaaaaiaaxMaacaWLjaWaaeWaceaacqaI3aWnaiaawIcacaGLPaaaaaa@4D91@

where *N*_*j *_is the total number of aphids on the source plant on day *j *and *K*_*p*_, is the carrying capacity of the plant. Dispersal rates and resource depletion were not considered. Each of the eight target plants was observed daily, and the number of apterous or winged aphids on each leaf was determined. All the aphids found on the target plants were removed, making it possible to measure the daily flow of insects arriving from the source plant. These estimates of dispersal did not take into account the aphids that may have dispersed beyond the target plants. The experiment was stopped 25 days after infestation.

### Optimality criteria and simulations

Dispersal strategies conferring maximum individual fitness in a clonal species such as *Aphis gossypii *should generate both the maximum number of aphids in the crop and the maximum number of winged propagules leaving the crop to colonize other fields. We considered both these criteria: the maximum number of aphids in the crop was determined as the maximum number of aphids present in the field during the crop season (*n*_*max*_). We roughly estimated the number of propagules (*n*_*prop*_) as the number of winged dispersers produced that did not land in the field. This number probably greatly overestimated the number of efficient propagules because it included individuals that died in the field or during dispersal. However, no data are available for precise estimation of the number of aphids that successfully disperse.

Of course, both fitness criteria are correlated. For instance, increasing the basic rate of flying dispersal (*A*_*f*_) obviously increases the number of propagules, and it decreases the maximal abundance because propagules are lost for the system. However, these fitness criteria describe the consequences of the balance between local population growth and dispersal.

We ran simulations of population dynamics in the field to test the effect of dispersal parameters on optimality criteria. The four parameters *A*_*w*_, *B*_*w*_, *A*_*f *_and *B*_*f *_were tested independently using 10 simulations for each of 21 values of the parameter. No simple rule of any biological significance could be used to define their range of variation, except that *A*_*w *_and *A*_*f *_have to be ≥ 0. Preliminary sets of simulations were run with different ranges, and the ranges describing the patterns of fitness variation and including observed values of the parameter and the values giving maximum or minimum fitness, when observed, were chosen. Potential effect on fitness of dispersal distance and leaf attractivity were then tested using 10 simulations of 21 values of *d *varying from 0 to 2, *f*_*at *_from 0 to 1 and *a *from 1 to 30.

Simulation models were based on a stochastic dispersal of walking and flying individuals. Changes in numbers were simulated using random assignments to integer values. Thus, results differed each time a simulation was done, even with the same set of parameters. Running 10 simulations with each parameter set was necessary to separate variations due to stochasticity from variations due to parameter values. Stochastic variations in the simulation results are illustrated by the standard deviations in figures.

## Authors' contributions

EL and LL conceived the study. EL and RB collected the biological data. EL performed the statistical analyses and wrote the paper. LL designed the model, performed the simulations and wrote part of the paper. All authors read and approved the final manuscript.
